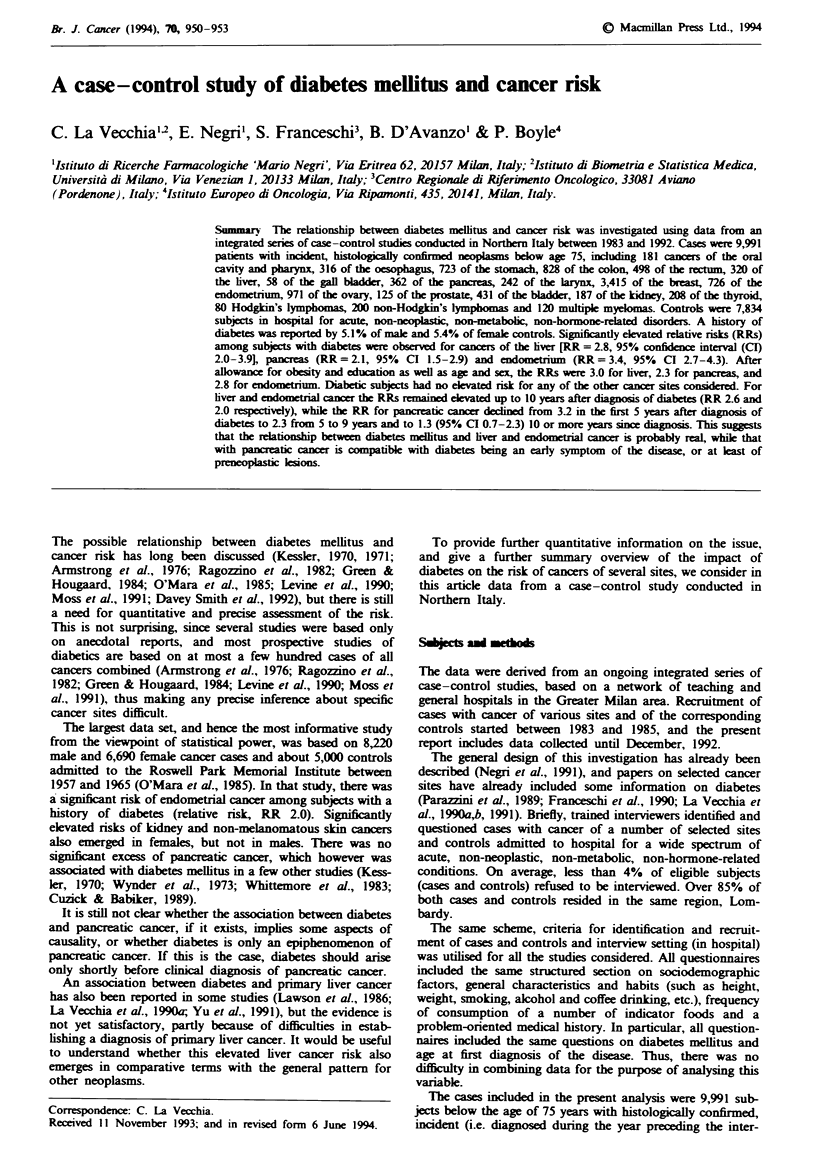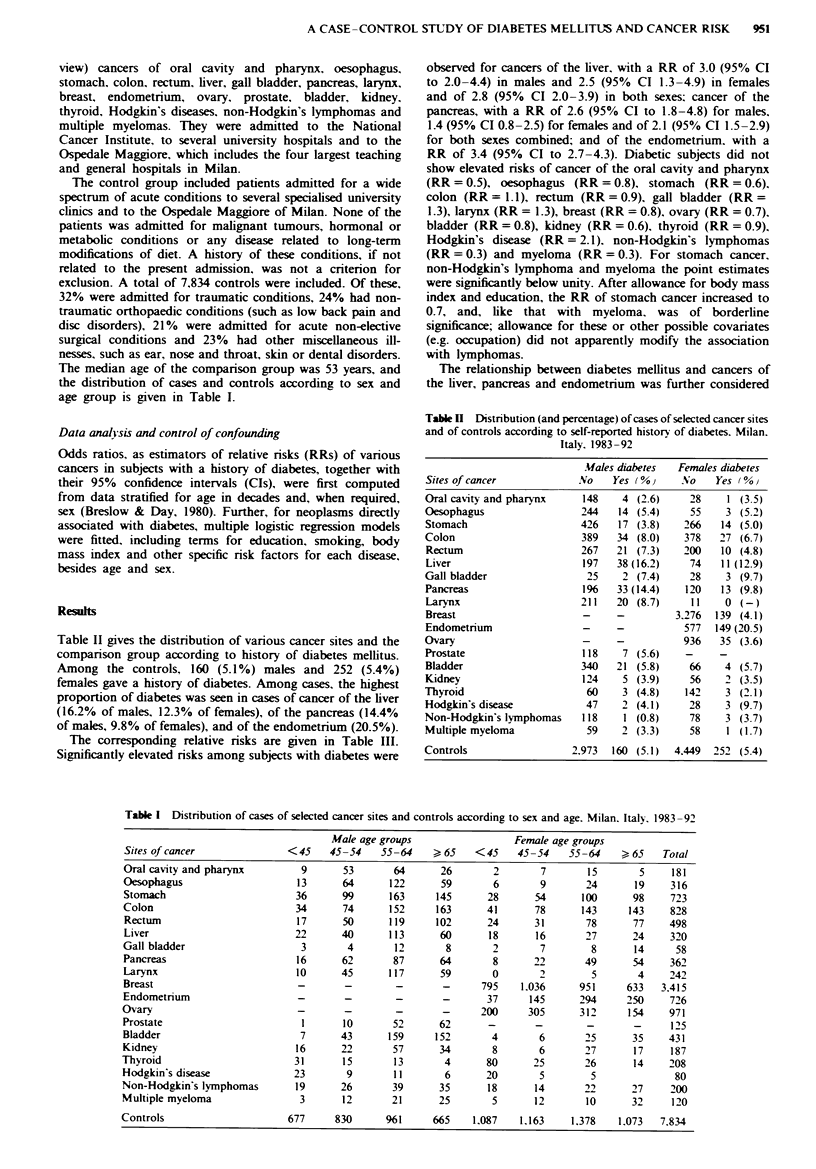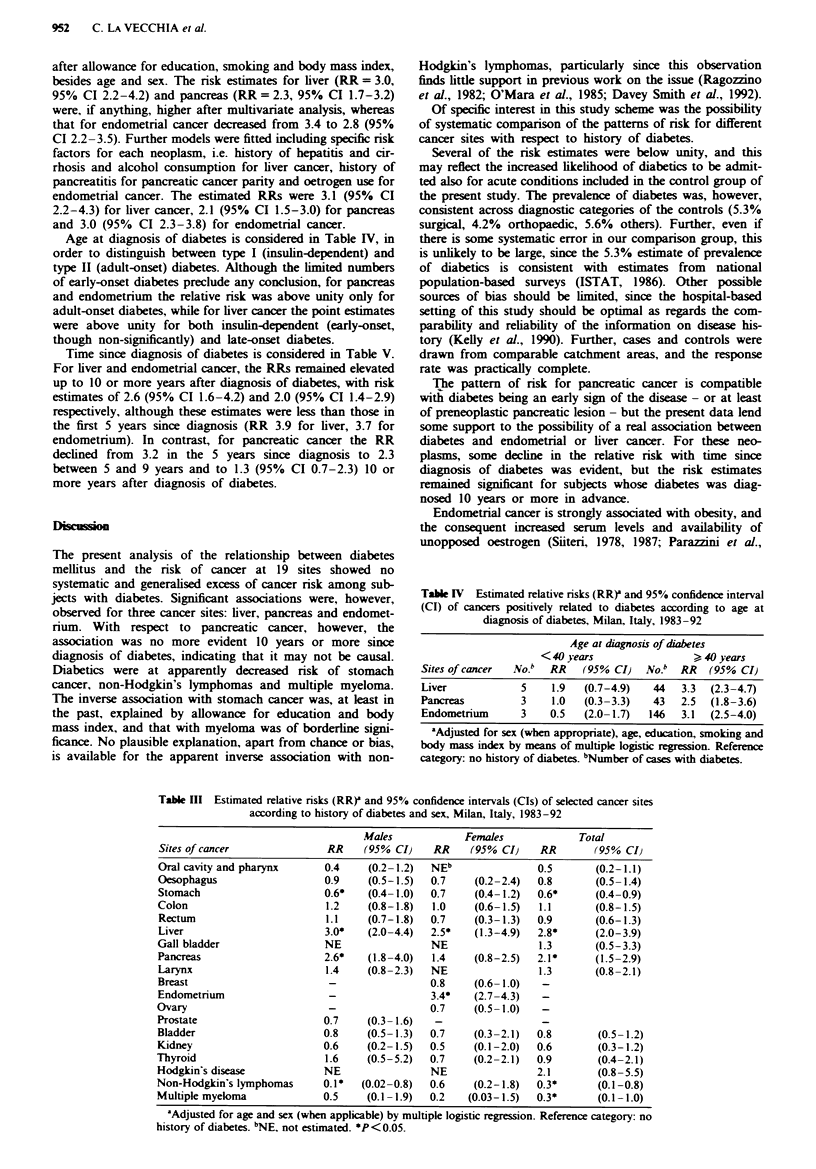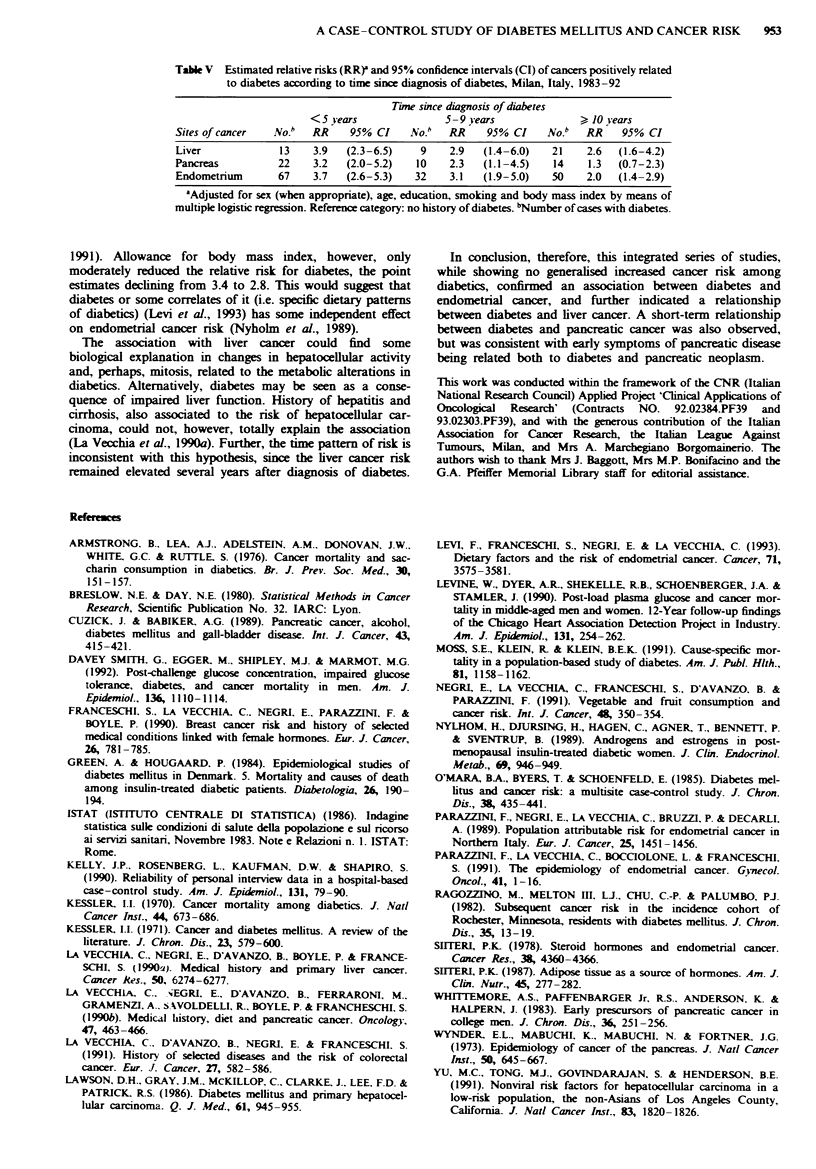# A case-control study of diabetes mellitus and cancer risk.

**DOI:** 10.1038/bjc.1994.427

**Published:** 1994-11

**Authors:** C. La Vecchia, E. Negri, S. Franceschi, B. D'Avanzo, P. Boyle

**Affiliations:** Istituto di Ricerche Farmacologiche Mario Negri, Milan, Italy.

## Abstract

The relationship between diabetes mellitus and cancer risk was investigated using data from an integrated series of case-control studies conducted in Northern Italy between 1983 and 1992. Cases were 9,991 patients with incident, histologically confirmed neoplasms below age 75, including 181 cancers of the oral cavity and pharynx, 316 of the oesophagus, 723 of the stomach, 828 of the colon, 498 of the rectum, 320 of the liver, 58 of the gall bladder, 362 of the pancreas, 242 of the larynx, 3,415 of the breast, 726 of the endometrium, 971 of the ovary, 125 of the prostate, 431 of the bladder, 187 of the kidney, 208 of the thyroid, 80 Hodgkin's lymphomas, 200 non-Hodgkin's lymphomas and 120 multiple myelomas. Controls were 7,834 subjects in hospital for acute, non-neoplastic, non-metabolic, non-hormone-related disorders. A history of diabetes was reported by 5.1% of male and 5.4% of female controls. Significantly elevated relative risks (RRs) among subjects with diabetes were observed for cancers of the liver [RR = 2.8, 95% confidence interval (CI) 2.0-3.9], pancreas (RR = 2.1, 95% CI 1.5-2.9) and endometrium (RR 3.4, 95% CI 2.7-4.3). After allowance for obesity and education as well as age and sex, the RRs were 3.0 for liver, 2.3 for pancreas, and 2.8 for endometrium. Diabetic subjects had no elevated risk for any of the other cancer sites considered. For liver and endometrial cancer the RRs remained elevated up to 10 years after diagnosis of diabetes (RR 2.6 and 2.0 respectively), while the RR for pancreatic cancer declined from 3.2 in the first 5 years after diagnosis of diabetes to 2.3 from 5 to 9 years and to 1.3 (95% CI 0.7-2.3) 10 or more years since diagnosis. This suggests that the relationship between diabetes mellitus and liver and endometrial cancer is probably real, while that with pancreatic cancer is compatible with diabetes being an early symptom of the disease, or at least of preneoplastic lesions.


Br. J. Cancer (1994), 70, 950-953 © Macmillan Press Ltd., 1994

### A case-control study of diabetes mellitus and cancer risk

C. La Vecchia[1,2], E. Negri[1], S. Franceschi[3], B. D'Avanzo[1] & P. Boyle[4]

[1]*Istituto di Ricerche Farmacologiche 'Mario Negri', Via Eritrea 62, 20157 Milan, Italy;* [2]*Istituto di Biometria e Statistica Medica, Università di Milano, Via Venezian 1, 20133 Milan, Italy;* [3]*Centro Regionale di Riferimento Oncologico, 33081 Aviano (Pordenone), Italy;* [4]*Istituto Europeo di Oncologia, Via Ripamonti, 435, 20141, Milan, Italy.*

**Summary** The relationship between diabetes mellitus and cancer risk was investigated using data from an integrated series of case-control studies conducted in Northern Italy between 1983 and 1992. Cases were 9,991 patients with incident, histologically confirmed neoplasms below age 75, including 181 cancers of the oral cavity and pharynx, 316 of the oesophagus, 723 of the stomach, 828 of the colon, 498 of the rectum, 320 of the liver, 58 of the gall bladder, 362 of the pancreas, 242 of the larynx, 3,415 of the breast, 726 of the endometrium, 971 of the ovary, 125 of the prostate, 431 of the bladder, 187 of the kidney, 208 of the thyroid, 80 Hodgkin's lymphomas, 200 non-Hodgkin's lymphomas and 120 multiple myelomas. Controls were 7,834 subjects in hospital for acute, non-neoplastic, non-metabolic, non-hormone-related disorders. A history of diabetes was reported by 5.1% of male and 5.4% of female controls. Significantly elevated relative risks (RRs) among subjects with diabetes were observed for cancers of the liver [RR = 2.8, 95% confidence interval (CI) 2.0-3.9], pancreas (RR = 2.1, 95% CI 1.5-2.9) and endometrium (RR = 3.4, 95% CI 2.7-4.3). After allowance for obesity and education as well as age and sex, the RRs were 3.0 for liver, 2.3 for pancreas, and 2.8 for endometrium. Diabetic subjects had no elevated risk for any of the other cancer sites considered. For liver and endometrial cancer the RRs remained elevated up to 10 years after diagnosis of diabetes (RR 2.6 and 2.0 respectively), while the RR for pancreatic cancer declined from 3.2 in the first 5 years after diagnosis of diabetes to 2.3 from 5 to 9 years and to 1.3 (95% CI 0.7-2.3) 10 or more years since diagnosis. This suggests that the relationship between diabetes mellitus and liver and endometrial cancer is probably real, while that with pancreatic cancer is compatible with diabetes being an early symptom of the disease, or at least of preneoplastic lesions.

The possible relationship between diabetes mellitus and cancer risk has long been discussed (Kessler, 1970, 1971; Armstrong *et al.*, 1976; Ragozzino *et al.*, 1982; Green & Hougaard, 1984; O'Mara *et al.*, 1985; Levine *et al.*, 1990; Moss *et al.*, 1991; Davey Smith *et al.*, 1992), but there is still a need for quantitative and precise assessment of the risk. This is not surprising, since several studies were based only on anecdotal reports, and most prospective studies of diabetics are based on at most a few hundred cases of all cancers combined (Armstrong *et al.*, 1976; Ragozzino *et al.*, 1982; Green & Hougaard, 1984; Levine *et al.*, 1990; Moss *et al.*, 1991), thus making any precise inference about specific cancer sites difficult.

The largest data set, and hence the most informative study from the viewpoint of statistical power, was based on 8,220 male and 6,690 female cancer cases and about 5,000 controls admitted to the Roswell Park Memorial Institute between 1957 and 1965 (O'Mara *et al.*, 1985). In that study, there was a significant risk of endometrial cancer among subjects with a history of diabetes (relative risk, RR 2.0). Significantly elevated risks of kidney and non-melanomatous skin cancers also emerged in females, but not in males. There was no significant excess of pancreatic cancer, which however was associated with diabetes mellitus in a few other studies (Kessler, 1970; Wynder *et al.*, 1973; Whittemore *et al.*, 1983; Cuzick & Babiker, 1989).

It is still not clear whether the association between diabetes and pancreatic cancer, if it exists, implies some aspects of causality, or whether diabetes is only an epiphenomenon of pancreatic cancer. If this is the case, diabetes should arise only shortly before clinical diagnosis of pancreatic cancer.

An association between diabetes and primary liver cancer has also been reported in some studies (Lawson *et al.*, 1986; La Vecchia *et al.*, 1990*a*; Yu *et al.*, 1991), but the evidence is not yet satisfactory, partly because of difficulties in establishing a diagnosis of primary liver cancer. It would be useful to understand whether this elevated liver cancer risk also emerges in comparative terms with the general pattern for other neoplasms.

Correspondence: C. La Vecchia.
Received 11 November 1993; and in revised form 6 June 1994.

To provide further quantitative information on the issue, and give a further summary overview of the impact of diabetes on the risk of cancers of several sites, we consider in this article data from a case-control study conducted in Northern Italy.

### Subjects and methods

The data were derived from an ongoing integrated series of case-control studies, based on a network of teaching and general hospitals in the Greater Milan area. Recruitment of cases with cancer of various sites and of the corresponding controls started between 1983 and 1985, and the present report includes data collected until December, 1992.

The general design of this investigation has already been described (Negri *et al.*, 1991), and papers on selected cancer sites have already included some information on diabetes (Parazzini *et al.*, 1989; Franceschi *et al.*, 1990; La Vecchia *et al.*, 1990*a*,*b*, 1991). Briefly, trained interviewers identified and questioned cases with cancer of a number of selected sites and controls admitted to hospital for a wide spectrum of acute, non-neoplastic, non-metabolic, non-hormone-related conditions. On average, less than 4% of eligible subjects (cases and controls) refused to be interviewed. Over 85% of both cases and controls resided in the same region, Lombardy.

The same scheme, criteria for identification and recruitment of cases and controls and interview setting (in hospital) was utilised for all the studies considered. All questionnaires included the same structured section on sociodemographic factors, general characteristics and habits (such as height, weight, smoking, alcohol and coffee drinking, etc.), frequency of consumption of a number of indicator foods and a problem-oriented medical history. In particular, all questionnaires included the same questions on diabetes mellitus and age at first diagnosis of the disease. Thus, there was no difficulty in combining data for the purpose of analysing this variable.

The cases included in the present analysis were 9,991 subjects below the age of 75 years with histologically confirmed, incident (i.e. diagnosed during the year preceding the interview) cancers of oral cavity and pharynx, oesophagus, stomach, colon, rectum, liver, gall bladder, pancreas, larynx, breast, endometrium, ovary, prostate, bladder, kidney, thyroid, Hodgkin's diseases, non-Hodgkin's lymphomas and multiple myelomas. They were admitted to the National Cancer Institute, to several university hospitals and to the Ospedale Maggiore, which includes the four largest teaching and general hospitals in Milan.

The control group included patients admitted for a wide spectrum of acute conditions to several specialised university clinics and to the Ospedale Maggiore of Milan. None of the patients was admitted for malignant tumours, hormonal or metabolic conditions or any disease related to long-term modifications of diet. A history of these conditions, if not related to the present admission, was not a criterion for exclusion. A total of 7,834 controls were included. Of these, 32% were admitted for traumatic conditions, 24% had non-traumatic orthopaedic conditions (such as low back pain and disc disorders), 21% were admitted for acute non-elective surgical conditions and 23% had other miscellaneous illnesses, such as ear, nose and throat, skin or dental disorders. The median age of the comparison group was 53 years, and the distribution of cases and controls according to sex and age group is given in Table I.

### *Data analysis and control of confounding*

Odds ratios, as estimators of relative risks (RRs) of various cancers in subjects with a history of diabetes, together with their 95% confidence intervals (CIs), were first computed from data stratified for age in decades and, when required, sex (Breslow & Day, 1980). Further, for neoplasms directly associated with diabetes, multiple logistic regression models were fitted, including terms for education, smoking, body mass index and other specific risk factors for each disease, besides age and sex.

### Results

Table II gives the distribution of various cancer sites and the comparison group according to history of diabetes mellitus. Among the controls, 160 (5.1%) males and 252 (5.4%) females gave a history of diabetes. Among cases, the highest proportion of diabetes was seen in cases of cancer of the liver (16.2% of males, 12.3% of females), of the pancreas (14.4% of males, 9.8% of females), and of the endometrium (20.5%).

The corresponding relative risks are given in Table III. Significantly elevated risks among subjects with diabetes were observed for cancers of the liver, with a RR of 3.0 (95% CI to 2.0–4.4) in males and 2.5 (95% CI 1.3–4.9) in females and of 2.8 (95% CI 2.0–3.9) in both sexes; cancer of the pancreas, with a RR of 2.6 (95% CI to 1.8–4.8) for males, 1.4 (95% CI 0.8–2.5) for females and of 2.1 (95% CI 1.5–2.9) for both sexes combined; and of the endometrium, with a RR of 3.4 (95% CI to 2.7–4.3). Diabetic subjects did not show elevated risks of cancer of the oral cavity and pharynx (RR = 0.5), oesophagus (RR = 0.8), stomach (RR = 0.6), colon (RR = 1.1), rectum (RR = 0.9), gall bladder (RR = 1.3), larynx (RR = 1.3), breast (RR = 0.8), ovary (RR = 0.7), bladder (RR = 0.8), kidney (RR = 0.6), thyroid (RR = 0.9), Hodgkin's disease (RR = 2.1), non-Hodgkin's lymphomas (RR = 0.3) and myeloma (RR = 0.3). For stomach cancer, non-Hodgkin's lymphoma and myeloma the point estimates were significantly below unity. After allowance for body mass index and education, the RR of stomach cancer increased to 0.7, and, like that with myeloma, was of borderline significance; allowance for these or other possible covariates (e.g. occupation) did not apparently modify the association with lymphomas.

The relationship between diabetes mellitus and cancers of the liver, pancreas and endometrium was further considered

**Table II** Distribution (and percentage) of cases of selected cancer sites and of controls according to self-reported history of diabetes, Milan, Italy, 1983–92

| Sites of cancer | Males diabetes No | Males diabetes Yes (%) | Females diabetes No | Females diabetes Yes (%) |
|---|---|---|---|---|
| Oral cavity and pharynx | 148 | 4 (2.6) | 28 | 1 (3.5) |
| Oesophagus | 244 | 14 (5.4) | 55 | 3 (5.2) |
| Stomach | 426 | 17 (3.8) | 266 | 14 (5.0) |
| Colon | 389 | 34 (8.0) | 378 | 27 (6.7) |
| Rectum | 267 | 21 (7.3) | 200 | 10 (4.8) |
| Liver | 197 | 38 (16.2) | 74 | 11 (12.9) |
| Gall bladder | 25 | 2 (7.4) | 28 | 3 (9.7) |
| Pancreas | 196 | 33 (14.4) | 120 | 13 (9.8) |
| Larynx | 211 | 20 (8.7) | 11 | 0 (–) |
| Breast | – | – | 3,276 | 139 (4.1) |
| Endometrium | – | – | 577 | 149 (20.5) |
| Ovary | – | – | 936 | 35 (3.6) |
| Prostate | 118 | 7 (5.6) | – | – |
| Bladder | 340 | 21 (5.8) | 66 | 4 (5.7) |
| Kidney | 124 | 5 (3.9) | 56 | 2 (3.5) |
| Thyroid | 60 | 3 (4.8) | 142 | 3 (2.1) |
| Hodgkin's disease | 47 | 2 (4.1) | 28 | 3 (9.7) |
| Non-Hodgkin's lymphomas | 118 | 1 (0.8) | 78 | 3 (3.7) |
| Multiple myeloma | 59 | 2 (3.3) | 58 | 1 (1.7) |
| Controls | 2,973 | 160 (5.1) | 4,449 | 252 (5.4) |

**Table I** Distribution of cases of selected cancer sites and controls according to sex and age, Milan, Italy, 1983–92

| Sites of cancer | Male age groups <45 | 45–54 | 55–64 | ≥65 | Female age groups <45 | 45–54 | 55–64 | ≥65 | Total |
|---|---|---|---|---|---|---|---|---|---|
| Oral cavity and pharynx | 9 | 53 | 64 | 26 | 2 | 7 | 15 | 5 | 181 |
| Oesophagus | 13 | 64 | 122 | 59 | 6 | 9 | 24 | 19 | 316 |
| Stomach | 36 | 99 | 163 | 145 | 28 | 54 | 100 | 98 | 723 |
| Colon | 34 | 74 | 152 | 163 | 41 | 78 | 143 | 143 | 828 |
| Rectum | 17 | 50 | 119 | 102 | 24 | 31 | 78 | 77 | 498 |
| Liver | 22 | 40 | 113 | 60 | 18 | 16 | 27 | 24 | 320 |
| Gall bladder | 3 | 4 | 12 | 8 | 2 | 7 | 8 | 14 | 58 |
| Pancreas | 16 | 62 | 87 | 64 | 8 | 22 | 49 | 54 | 362 |
| Larynx | 10 | 45 | 117 | 59 | 0 | 2 | 5 | 4 | 242 |
| Breast | – | – | – | – | 795 | 1,036 | 951 | 633 | 3,415 |
| Endometrium | – | – | – | – | 37 | 145 | 294 | 250 | 726 |
| Ovary | – | – | – | – | 200 | 305 | 312 | 154 | 971 |
| Prostate | 1 | 10 | 52 | 62 | – | – | – | – | 125 |
| Bladder | 7 | 43 | 159 | 152 | 4 | 6 | 25 | 35 | 431 |
| Kidney | 16 | 22 | 57 | 34 | 8 | 6 | 27 | 17 | 187 |
| Thyroid | 31 | 15 | 13 | 4 | 80 | 25 | 26 | 14 | 208 |
| Hodgkin's disease | 23 | 9 | 11 | 6 | 20 | 5 | 5 | | 80 |
| Non-Hodgkin's lymphomas | 19 | 26 | 39 | 35 | 18 | 14 | 22 | 27 | 200 |
| Multiple myeloma | 3 | 12 | 21 | 25 | 5 | 12 | 10 | 32 | 120 |
| Controls | 677 | 830 | 961 | 665 | 1,087 | 1,163 | 1,378 | 1,073 | 7,834 |

after allowance for education, smoking and body mass index, besides age and sex. The risk estimates for liver (RR = 3.0, 95% CI 2.2–4.2) and pancreas (RR = 2.3, 95% CI 1.7–3.2) were, if anything, higher after multivariate analysis, whereas that for endometrial cancer decreased from 3.4 to 2.8 (95% CI 2.2–3.5). Further models were fitted including specific risk factors for each neoplasm, i.e. history of hepatitis and cirrhosis and alcohol consumption for liver cancer, history of pancreatitis for pancreatic cancer parity and oetrogen use for endometrial cancer. The estimated RRs were 3.1 (95% CI 2.2–4.3) for liver cancer, 2.1 (95% CI 1.5–3.0) for pancreas and 3.0 (95% CI 2.3–3.8) for endometrial cancer.

Age at diagnosis of diabetes is considered in Table IV, in order to distinguish between type I (insulin-dependent) and type II (adult-onset) diabetes. Although the limited numbers of early-onset diabetes preclude any conclusion, for pancreas and endometrium the relative risk was above unity only for adult-onset diabetes, while for liver cancer the point estimates were above unity for both insulin-dependent (early-onset, though non-significantly) and late-onset diabetes.

Time since diagnosis of diabetes is considered in Table V. For liver and endometrial cancer, the RRs remained elevated up to 10 or more years after diagnosis of diabetes, with risk estimates of 2.6 (95% CI 1.6–4.2) and 2.0 (95% CI 1.4–2.9) respectively, although these estimates were less than those in the first 5 years since diagnosis (RR 3.9 for liver, 3.7 for endometrium). In contrast, for pancreatic cancer the RR declined from 3.2 in the 5 years since diagnosis to 2.3 between 5 and 9 years and to 1.3 (95% CI 0.7–2.3) 10 or more years after diagnosis of diabetes.

### Discussion

The present analysis of the relationship between diabetes mellitus and the risk of cancer at 19 sites showed no systematic and generalised excess of cancer risk among subjects with diabetes. Significant associations were, however, observed for three cancer sites: liver, pancreas and endometrium. With respect to pancreatic cancer, however, the association was no more evident 10 years or more since diagnosis of diabetes, indicating that it may not be causal. Diabetics were at apparently decreased risk of stomach cancer, non-Hodgkin's lymphomas and multiple myeloma. The inverse association with stomach cancer was, at least in the past, explained by allowance for education and body mass index, and that with myeloma was of borderline significance. No plausible explanation, apart from chance or bias, is available for the apparent inverse association with non-Hodgkin's lymphomas, particularly since this observation finds little support in previous work on the issue (Ragozzino et al., 1982; O'Mara et al., 1985; Davey Smith et al., 1992).

Of specific interest in this study scheme was the possibility of systematic comparison of the patterns of risk for different cancer sites with respect to history of diabetes.

Several of the risk estimates were below unity, and this may reflect the increased likelihood of diabetics to be admitted also for acute conditions included in the control group of the present study. The prevalence of diabetes was, however, consistent across diagnostic categories of the controls (5.3% surgical, 4.2% orthopaedic, 5.6% others). Further, even if there is some systematic error in our comparison group, this is unlikely to be large, since the 5.3% estimate of prevalence of diabetics is consistent with estimates from national population-based surveys (ISTAT, 1986). Other possible sources of bias should be limited, since the hospital-based setting of this study should be optimal as regards the comparability and reliability of the information on disease history (Kelly et al., 1990). Further, cases and controls were drawn from comparable catchment areas, and the response rate was practically complete.

The pattern of risk for pancreatic cancer is compatible with diabetes being an early sign of the disease – or at least of preneoplastic pancreatic lesion – but the present data lend some support to the possibility of a real association between diabetes and endometrial or liver cancer. For these neoplasms, some decline in the relative risk with time since diagnosis of diabetes was evident, but the risk estimates remained significant for subjects whose diabetes was diagnosed 10 years or more in advance.

Endometrial cancer is strongly associated with obesity, and the consequent increased serum levels and availability of unopposed oestrogen (Siiteri, 1978, 1987; Parazzini et al.,

**Table IV** Estimated relative risks (RR)[a] and 95% confidence interval (CI) of cancers positively related to diabetes according to age at diagnosis of diabetes, Milan, Italy, 1983–92

| | Age at diagnosis of diabetes | | | | | |
|---|---|---|---|---|---|---|
| | < 40 years | | | ⩾ 40 years | | |
| Sites of cancer | No.[b] | RR | (95% CI) | No.[b] | RR | (95% CI) |
| Liver | 5 | 1.9 | (0.7–4.9) | 44 | 3.3 | (2.3–4.7) |
| Pancreas | 3 | 1.0 | (0.3–3.3) | 43 | 2.5 | (1.8–3.6) |
| Endometrium | 3 | 0.5 | (2.0–1.7) | 146 | 3.1 | (2.5–4.0) |

[a]Adjusted for sex (when appropriate), age, education, smoking and body mass index by means of multiple logistic regression. Reference category: no history of diabetes. [b]Number of cases with diabetes.

**Table III** Estimated relative risks (RR)[a] and 95% confidence intervals (CIs) of selected cancer sites according to history of diabetes and sex, Milan, Italy, 1983–92

| | Males | | Females | | Total | |
|---|---|---|---|---|---|---|
| Sites of cancer | RR | (95% CI) | RR | (95% CI) | RR | (95% CI) |
| Oral cavity and pharynx | 0.4 | (0.2–1.2) | NE[b] | | 0.5 | (0.2–1.1) |
| Oesophagus | 0.9 | (0.5–1.5) | 0.7 | (0.2–2.4) | 0.8 | (0.5–1.4) |
| Stomach | 0.6* | (0.4–1.0) | 0.7 | (0.4–1.2) | 0.6* | (0.4–0.9) |
| Colon | 1.2 | (0.8–1.8) | 1.0 | (0.6–1.5) | 1.1 | (0.8–1.5) |
| Rectum | 1.1 | (0.7–1.8) | 0.7 | (0.3–1.3) | 0.9 | (0.6–1.3) |
| Liver | 3.0* | (2.0–4.4) | 2.5* | (1.3–4.9) | 2.8* | (2.0–3.9) |
| Gall bladder | NE | | NE | | 1.3 | (0.5–3.3) |
| Pancreas | 2.6* | (1.8–4.0) | 1.4 | (0.8–2.5) | 2.1* | (1.5–2.9) |
| Larynx | 1.4 | (0.8–2.3) | NE | | 1.3 | (0.8–2.1) |
| Breast | – | | 0.8 | (0.6–1.0) | – | |
| Endometrium | – | | 3.4* | (2.7–4.3) | – | |
| Ovary | – | | 0.7 | (0.5–1.0) | – | |
| Prostate | 0.7 | (0.3–1.6) | – | | – | |
| Bladder | 0.8 | (0.5–1.3) | 0.7 | (0.3–2.1) | 0.8 | (0.5–1.2) |
| Kidney | 0.6 | (0.2–1.5) | 0.5 | (0.1–2.0) | 0.6 | (0.3–1.2) |
| Thyroid | 1.6 | (0.5–5.2) | 0.7 | (0.2–2.1) | 0.9 | (0.4–2.1) |
| Hodgkin's disease | NE | | NE | | 2.1 | (0.8–5.5) |
| Non-Hodgkin's lymphomas | 0.1* | (0.02–0.8) | 0.6 | (0.2–1.8) | 0.3* | (0.1–0.8) |
| Multiple myeloma | 0.5 | (0.1–1.9) | 0.2 | (0.03–1.5) | 0.3* | (0.1–1.0) |

[a]Adjusted for age and sex (when applicable) by multiple logistic regression. Reference category: no history of diabetes. [b]NE, not estimated. *$P<0.05$.

**Table V** Estimated relative risks (RR)[a] and 95% confidence intervals (CI) of cancers positively related to diabetes according to time since diagnosis of diabetes, Milan, Italy, 1983–92

| | *Time since diagnosis of diabetes* | | | | | | | | |
|---|---|---|---|---|---|---|---|---|---|
| | *<5 years* | | | *5–9 years* | | | *≥ 10 years* | | |
| *Sites of cancer* | *No.*[b] | *RR* | *95% CI* | *No.*[b] | *RR* | *95% CI* | *No.*[b] | *RR* | *95% CI* |
| Liver | 13 | 3.9 | (2.3–6.5) | 9 | 2.9 | (1.4–6.0) | 21 | 2.6 | (1.6–4.2) |
| Pancreas | 22 | 3.2 | (2.0–5.2) | 10 | 2.3 | (1.1–4.5) | 14 | 1.3 | (0.7–2.3) |
| Endometrium | 67 | 3.7 | (2.6–5.3) | 32 | 3.1 | (1.9–5.0) | 50 | 2.0 | (1.4–2.9) |

[a]Adjusted for sex (when appropriate), age, education, smoking and body mass index by means of multiple logistic regression. Reference category: no history of diabetes. [b]Number of cases with diabetes.

1991). Allowance for body mass index, however, only moderately reduced the relative risk for diabetes, the point estimates declining from 3.4 to 2.8. This would suggest that diabetes or some correlates of it (i.e. specific dietary patterns of diabetics) (Levi *et al.*, 1993) has some independent effect on endometrial cancer risk (Nyholm *et al.*, 1989).

The association with liver cancer could find some biological explanation in changes in hepatocellular activity and, perhaps, mitosis, related to the metabolic alterations in diabetics. Alternatively, diabetes may be seen as a consequence of impaired liver function. History of hepatitis and cirrhosis, also associated to the risk of hepatocellular carcinoma, could not, however, totally explain the association (La Vecchia *et al.*, 1990*a*). Further, the time pattern of risk is inconsistent with this hypothesis, since the liver cancer risk remained elevated several years after diagnosis of diabetes.

In conclusion, therefore, this integrated series of studies, while showing no generalised increased cancer risk among diabetics, confirmed an association between diabetes and endometrial cancer, and further indicated a relationship between diabetes and liver cancer. A short-term relationship between diabetes and pancreatic cancer was also observed, but was consistent with early symptoms of pancreatic disease being related both to diabetes and pancreatic neoplasm.

This work was conducted within the framework of the CNR (Italian National Research Council) Applied Project 'Clinical Applications of Oncological Research' (Contracts NO. 92.02384.PF39 and 93.02303.PF39), and with the generous contribution of the Italian Association for Cancer Research, the Italian League Against Tumours, Milan, and Mrs A. Marchegiano Borgomainerio. The authors wish to thank Mrs J. Baggott, Mrs M.P. Bonifacino and the G.A. Pfeiffer Memorial Library staff for editorial assistance.

### References

ARMSTRONG, B., LEA, A.J., ADELSTEIN, A.M., DONOVAN, J.W., WHITE, G.C. & RUTTLE, S. (1976). Cancer mortality and saccharin consumption in diabetics. *Br. J. Prev. Soc. Med.*, **30**, 151–157.

BRESLOW, N.E. & DAY, N.E. (1980). *Statistical Methods in Cancer Research*, Scientific Publication No. 32. IARC: Lyon.

CUZICK, J. & BABIKER, A.G. (1989). Pancreatic cancer, alcohol, diabetes mellitus and gall-bladder disease. *Int. J. Cancer*, **43**, 415–421.

DAVEY SMITH, G., EGGER, M., SHIPLEY, M.J. & MARMOT, M.G. (1992). Post-challenge glucose concentration, impaired glucose tolerance, diabetes, and cancer mortality in men. *Am. J. Epidemiol.*, **136**, 1110–1114.

FRANCESCHI, S., LA VECCHIA, C., NEGRI, E., PARAZZINI, F. & BOYLE, P. (1990). Breast cancer risk and history of selected medical conditions linked with female hormones. *Eur. J. Cancer*, **26**, 781–785.

GREEN, A. & HOUGAARD, P. (1984). Epidemiological studies of diabetes mellitus in Denmark. 5. Mortality and causes of death among insulin-treated diabetic patients. *Diabetologia*, **26**, 190–194.

ISTAT (ISTITUTO CENTRALE DI STATISTICA) (1986). Indagine statistica sulle condizioni di salute della popolazione e sul ricorso ai servizi sanitari, Novembre 1983. Note e Relazioni n. 1. ISTAT: Rome.

KELLY, J.P., ROSENBERG, L., KAUFMAN, D.W. & SHAPIRO, S. (1990). Reliability of personal interview data in a hospital-based case-control study. *Am. J. Epidemiol.*, **131**, 79–90.

KESSLER, I.I. (1970). Cancer mortality among diabetics. *J. Natl Cancer Inst.*, **44**, 673–686.

KESSLER, I.I. (1971). Cancer and diabetes mellitus. A review of the literature. *J. Chron. Dis.*, **23**, 579–600.

LA VECCHIA, C., NEGRI, E., D'AVANZO, B., BOYLE, P. & FRANCESCHI, S. (1990*a*). Medical history and primary liver cancer. *Cancer Res.*, **50**, 6274–6277.

LA VECCHIA, C., NEGRI, E., D'AVANZO, B., FERRARONI, M., GRAMENZI, A., SAVOLDELLI, R., BOYLE, P. & FRANCHESCHI, S. (1990*b*). Medical history, diet and pancreatic cancer. *Oncology*, **47**, 463–466.

LA VECCHIA, C., D'AVANZO, B., NEGRI, E. & FRANCESCHI, S. (1991). History of selected diseases and the risk of colorectal cancer. *Eur. J. Cancer*, **27**, 582–586.

LAWSON, D.H., GRAY, J.M., MCKILLOP, C., CLARKE, J., LEE, F.D. & PATRICK, R.S. (1986). Diabetes mellitus and primary hepatocellular carcinoma. *Q. J. Med.*, **61**, 945–955.

LEVI, F., FRANCESCHI, S., NEGRI, E. & LA VECCHIA, C. (1993). Dietary factors and the risk of endometrial cancer. *Cancer*, **71**, 3575–3581.

LEVINE, W., DYER, A.R., SHEKELLE, R.B., SCHOENBERGER, J.A. & STAMLER, J. (1990). Post-load plasma glucose and cancer mortality in middle-aged men and women. 12-Year follow-up findings of the Chicago Heart Association Detection Project in Industry. *Am. J. Epidemiol.*, **131**, 254–262.

MOSS, S.E., KLEIN, R. & KLEIN, B.E.K. (1991). Cause-specific mortality in a population-based study of diabetes. *Am. J. Publ. Hlth.*, **81**, 1158–1162.

NEGRI, E., LA VECCHIA, C., FRANCESCHI, S., D'AVANZO, B. & PARAZZINI, F. (1991). Vegetable and fruit consumption and cancer risk. *Int. J. Cancer*, **48**, 350–354.

NYLHOM, H., DJURSING, H., HAGEN, C., AGNER, T., BENNETT, P. & SVENTRUP, B. (1989). Androgens and estrogens in postmenopausal insulin-treated diabetic women. *J. Clin. Endocrinol. Metab.*, **69**, 946–949.

O'MARA, B.A., BYERS, T. & SCHOENFELD, E. (1985). Diabetes mellitus and cancer risk: a multisite case-control study. *J. Chron. Dis.*, **38**, 435–441.

PARAZZINI, F., NEGRI, E., LA VECCHIA, C., BRUZZI, P. & DECARLI, A. (1989). Population attributable risk for endometrial cancer in Northern Italy. *Eur. J. Cancer*, **25**, 1451–1456.

PARAZZINI, F., LA VECCHIA, C., BOCCIOLONE, L. & FRANCESCHI, S. (1991). The epidemiology of endometrial cancer. *Gynecol. Oncol.*, **41**, 1–16.

RAGOZZINO, M., MELTON III, L.J., CHU, C.-P. & PALUMBO, P.J. (1982). Subsequent cancer risk in the incidence cohort of Rochester, Minnesota, residents with diabetes mellitus. *J. Chron. Dis.*, **35**, 13–19.

SIITERI, P.K. (1978). Steroid hormones and endometrial cancer. *Cancer Res.*, **38**, 4360–4366.

SIITERI, P.K. (1987). Adipose tissue as a source of hormones. *Am. J. Clin. Nutr.*, **45**, 277–282.

WHITTEMORE, A.S., PAFFENBARGER Jr, R.S., ANDERSON, K. & HALPERN, J. (1983). Early prescursors of pancreatic cancer in college men. *J. Chron. Dis.*, **36**, 251–256.

WYNDER, E.L., MABUCHI, K., MABUCHI, N. & FORTNER, J.G. (1973). Epidemiology of cancer of the pancreas. *J. Natl Cancer Inst.*, **50**, 645–667.

YU, M.C., TONG, M.J., GOVINDARAJAN, S. & HENDERSON, B.E. (1991). Nonviral risk factors for hepatocellular carcinoma in a low-risk population, the non-Asians of Los Angeles County, California. *J. Natl Cancer Inst.*, **83**, 1820–1826.